# Leadership for Innovation in Healthcare: An Exploration

**DOI:** 10.15171/ijhpm.2018.122

**Published:** 2018-12-17

**Authors:** Philip Weintraub, Martin McKee

**Affiliations:** Department of Public Health & Policy, London School of Hygiene and Tropical Medicine, London, UK.

**Keywords:** Leadership Styles, Health Innovation, Innovation Process, National Health Service

## Abstract

Although leadership has been studied extensively, most research has focused on the political and military spheres. More recent work has also examined the role of leadership in sectors such as manufacturing and technology, both areas where it is essential to encourage and nurture innovation. Yet, in the health sector, where innovation is now high on the policy agenda in many countries, there is a paucity of research on how leadership can foster a culture of innovation. In this perspective, written for those seeking to foster innovation in the health sector, we offer a narrative synthesis approach of eight theories and concepts that have been empirically shown to support innovation through all phases of the innovation process.

## Introduction

This perspective arose from experience conducting an empirical study of organisations created within the English National Health Service to foster innovation for patient benefit, such as new technologies and models of care. Our discussions with those in leadership roles in these organisations pointed to a desire for a succinct review of concepts and theories that could inform their thinking. This perspective seeks to provide such a review.

As the terms ‘leadership’ and ‘innovation’ are at the core of this perspective, it is essential to define them at the outset. For the present purposes, we have taken the definition of leadership set out by Yukl^1^ as *“the process of influencing others to understand and agree about what needs to be done and how to do it, and the process of facilitating individual and collective efforts to accomplish shared objectives.”* Innovation is defined as *“a broad set of activities involving the creation and implementation of concepts and products new to an organisation.”*^2^

Given these definitions, it is almost a cliché to say that health systems must constantly innovate. First they must respond to changing disease burdens. The advent of HIV/AIDS exemplified how a new condition can have wide-ranging implications for many aspects of healthcare, from confidentiality, to patient’s rights, infection control, and much else. The growth of frailty and multi-morbidity in ageing populations has different, but similarly extensive implications for how care is delivered. A second factor is technology, with new ways of diagnosing and treating patients, offering cures for conditions once rapidly fatal or, more often, transforming them into long term disorders. Finally, there are new models of care, involving changing professional roles and advances in communication and data processing, increasingly informed by evidence from health services research.

Much has been written about the process of innovation, examining how a need is identified and a solution developed and implemented. This literature has considered many issues, including the roles of differing stakeholders, for example in government and industry, and the incentives they face,^3^ organisational cultures that foster innovation,^4^ and factors that influence diffusion of innovation.^5^ Thus, in the health sector, propensity to innovate has been explained by the characteristics of the individuals involved, their organisations, and the context.^6^ There is also a wealth of literature on leadership of innovation, especially in the manufacturing and technology sectors. However, despite its need for continued innovation, there is a paucity of such literature from the health sector,^7^ with a study of National Institutes of Health (NIH) funded genetic researchers concluding that “*leadership and management roles in research have received scant empirical examination”*^8^ (p. 408). The pressing need to fill this gap in the literature was emphasized in a recent study by Lombardi et al.^9^ They describe an “*existential urgency*” to adopt radical innovations to transform the US health system, caused by diminishing federal dollars devoted to health research in the United States and what they see as a severe lack of knowledge among, in their work, Academic Health Organizations located in the Southeastern US.

It is important that we clarify who this perspective is written for. It concentrates on the interface between innovation and healthcare. It examines leadership *for* innovation in the health sector, in other words where the primary goal is innovation to achieve better health. As noted above, our experience suggests that this is a group who believe that, so far, their needs have often been overlooked.

These individuals work with, but differ from those who manage the organic process of innovation throughout health systems, where it takes place alongside other streams of work. Such innovations relate to adoption of products, such as new medicines or technologies, processes, such as new models of care, processes, such as tele-surgery, and paradigms, such as the many changes in clinical practice that followed from the introduction of computerised tomography or minimally invasive surgery.^10^ Such new ideas are constantly emerging and being developed by those who identify better ways of performing their roles. However, the promotion of innovation is not their primary task. Thus, to reiterate, our specific focus is on organisations whose primary purpose is promoting and implementing innovation across the health sector. Nor is it written for the academic community, who will seek a much more detailed review of the literature than is possible in a short piece such as this. And nor is it intended for those working in highly specialised areas, such as NHS Genomic Medicine Centres, where the target audience is very narrowly circumscribed.

The scope of the perspective is also, necessarily, circumscribed in terms of the literature that it draws most directly on. There is a very large body of literature on leadership and on innovation per se. Some of this relates to the characteristics of individual leaders, such as Great Man Theory^11^ or Trait Theory.^12^ Other writers have focused on leadership for change, which may but need not necessarily involve innovation, such as Lewin’s^13^ and Likert’s^14^ theories. Other work relates to achievement of organisational goals,^15,16^ which again is important for any organisation, but not specifically to the promotion of innovation.

Nor does it explore the extensive literature on theories of innovation, such as the debate on radical versus incremental innovation, ideas of creative destruction,^17^ the extent to which organisations focus inwards or outwards,^18^ the role of innovation of components or linkages,^19^ or the substantial research on diffusion of innovation.^20^ However, while not directly relevant to this perspective, this literature has informed our thinking.

## Research Question

Our question is “which theories and concepts have been shown to encourage, or inhibit, the ability to lead processes of innovation in the health sector?” We have approached this by means of narrative synthesis.^21^ We begin by listing a series of theories and concepts from the broader literature on leadership for innovation and then seek examples from the health sector, by means of a series of searches of electronic databases. Our primary focus is on empirical studies although, where relevant, we note the existence of selected others that are speculative, proposing but not testing theories and concepts. Our approach is exploratory and iterative, and while we have undertaken a structured search process to identify studies, we recognize that this is unlikely to be exhaustive given the very wide range of disciplines, publication outlets, and terminologies involved. In this respect, we recognise the impossibility of being exhaustive noted by Wisdom et al in their systematic review of innovation that “*did not necessarily include every paper published on the topic.”*^22^

The search strategy used to identify this literature is reported in detail in Supplementary file 1.

## Literature Review

### Innovation Process

We now discuss the various theories and concepts identified. We refer, as appropriate, to phases in the process of innovation. Several frameworks have been proposed, such as that by Smith and Kaluzny,^23^ which has four phases, awareness (recognition of a gap between expected and actual performance), identification (developing a solution to close this gap), implementation, and institutionalization, so that the solution becomes integrated into routine activities and the one that we have selected, by Tidd and Bessant, who also identified four phases, *Search, Select, Implement* and *Capture*.^10^ In the ‘*Search*” phase of the innovation process, leaders/managers need to establish clear pathways and processes to bring new ideas/opportunities to the attention of senior leadership. To ‘*Select’* among a continuous flow of ideas/opportunities, a health organisation must have a strategic plan to guide leaders/managers to select from what is often a choice among these myriad ideas/opportunities, most of which will seem, at least at first glance, plausible and which are based on the best of human intentions. In the ‘*Implementation’* phase, the idea/opportunity is brought into reality. This may be in the form of: (1) *Product Innovation* (eg, new drug therapy); (2) *Process Innovation* (which can range from new and complex models of care or even simple measures such as using volunteers to feed inpatients); (3) *Position Innovation* (eg, perform remote electronic monitoring of patient formerly admitted to hospital); and *Paradigm Innovation* (eg, patients driving idea/opportunity generation within the health organisation).^10^ To* ‘Capture’* the organisational benefits of an innovation strategy requires deployment of the accumulated leadership and management knowledge and administrative/technical skills acquired in the prior three innovation phases, plus the capacity to launch, spread and sustain the innovation. We also recognise that innovation may or may not be disruptive, with the former defined in a recent European Commission expert panel paper as radical innovations that displace older organisational structures, workforce, processes, products, services and technologies,^24^ as opposed to those that are incremental measures.

## Theories and Concepts

### Creating a Psychological Climate for Innovation

The psychological climate of an organisation has been invoked as a factor in its ability to be innovative.^22,25^ Weng et al proposed that a supportive organisation would encourage employees to turn “*creative ideas into innovative outputs*”^26^ and, in a study of 439 Taiwanese nurses in four hospitals, found that a supportive organisational climate encouraged creativity and innovative behavior.^27^ Jacobs et al examined psychological climate in a study of innovation and implementation by individual physicians participating in the US National Cancer Institute’s Community Clinical Oncology Program, finding that it could have a positive effect on the ‘Implementation’ phase by describing *“the direct relationship between implementation climate and implementation effectiveness”* as one of the most important findings (p.12).^25^ These findings suggest that leaders should seek to foster such a climate. However, a supportive psychological climate is not, on its own, a guarantee of success in innovation and requires that the attention of leaders/managers be devoted to both organisational and individual concerns (ie, ‘Context’ [eg, economic; political; and operating environment] and ‘Group Cohesiveness’). Helfrich et al describe a framework for the adoption of complex innovations to be used in health organisations^28^ based on one first studied in a manufacturing environment, for example by Klein and Sorra.^29^ Helfrich et al concluded that the successful implementation of complex innovations was dependent on “*management support and innovation-values fit, which contribute to an organizational ‘climate’ for implementation”* (p.279).

### Leader Member-Exchange

The leader–member exchange (LMX) theory focuses on the two-way relationship between leaders and each of their followers.^30^ The quality of these relationships influences subordinates’ behaviour.^31^ High quality relationships are based on trust and respect often extending beyond the scope of employment. In other words, the quality of the LMX relationship is gauged by the extent to which employees perceive their leadership is *“acting in their best interest, caring, supportive, loyal and reliable”* (p. 133). High quality LMX, when accompanied by empowerment and leadership support, has been found to be associated with greater effectiveness in all phases of the innovation process.^22,32,33^

A quantitative study of senior management teams in 27 hospitals the United Kingdom found that group processes, in particular the relationship between the leaders/managers and members of the organisations studied, was the main predictor of innovation, although the number of innovative individuals best predicted how radical the innovations would be. Resources beyond a minimum to accomplish a task did not predict innovation.^34^

### Social Capital

While LMX is concerned with dyads, the links between two individuals, a leader and a follower, social capital relates to the wider network of relationships among people who live and work in a particular society or organisation. There is a vast literature on this subject, which goes far beyond what can be included in this perspective, and which has been reviewed in detail elsewhere.^35^ Our focus is on the social capital that links leaders/managers through relational and network assets such healthcare purchasers and providers, academic organisations, governmental and related agencies, non-governmental organisations, and industry and professional groups.

A quantitative study in the United States surveyed 1978 respondents from a cross-section of manufacturing and service companies regarding their use of external network assets, providing empirical support for the proposition that learning through external networks disproportionately benefits conservative, risk-averse firms.^36^ The authors concluded that “*breakthrough innovation has long been associated with an Entrepreneurial Orientation (EO)”* (p. 13). Few organisations have “*the culture, capabilities, human resources, or financial resources to morph themselves into a strong EO culture. This research demonstrates …the ability to utilize market knowledge, ideas, and interpretations from external networks, provides a means for firms …to successfully innovate*” (p. 13). A quantitative study of 440 respondents from the manufacturing sector evaluated the impact of Social Capital on innovation using explanatory variables such as *“business network assets, information network assets, research network assets, participation assets and relational assets and one form of cognitive Social Capital (reciprocal trust)”* (p. 2). It concluded that these forms of Social Capital “*contribute to a larger extent to explain both the decision to innovate or not and the decision to undertake more or less radical innovations”*^37^ (p. 15).

Given these findings, it seems surprising that few studies have examined the link between social capital and innovation in the health sector. One, of those working in health promotion in German banks, did find a link between greater social capital and innovation.^38^ Another examined Taiwanese hospitals, finding that while social capital did explain some of the variation observed, this was explained by its link to institutional capabilities.^39^ The authors also speculated that high levels of internal social capital, at least in large organisations, might inhibit innovation by reducing engagement with external actors.^40^ Kyratsis et al in a qualitative study of technology adoption in twelve NHS Trusts, alluded to the importance of “*professional networks [that] were widely used and comprised practice-based peer-mediated information about the innovations’ relevance to the local setting*”^41^ (p. 7).

In summary, while there is evidence of the value of social capital in promoting innovation from other sectors, so far the evidence in the health sector is limited and, to some extent, contradictory. What does exist suggests that benefits are more likely to arise more from the bridging form of social capital, linking across organisations, than the bonding form, concerned with links within them.^42^

### Leadership Clarity

Leadership clarity (in relation to the roles of team members) was found to foster organisational innovation in a study of 3147 respondents in healthcare teams in the United Kingdom. This quantitative study focused on three types of healthcare teams: 98 primary healthcare teams (PHCTs), 113 community mental health teams (CMHTs), and 72 breast cancer care teams (BCTs). Self-report questionnaires were completed by 1156 respondents from 98 PHCTs, 1443 respondents from 113 CMHTs, and 548 respondents from the 72 BCTs. They sought respondents’ perceptions of team functioning, innovation, leadership, and effectiveness. The findings pointed to the importance of not just a single leader, but also to managers showing leadership at all levels of the organisation. Innovation was more likely to result when the organisation’s leaders/managers understood its objectives and where high levels of team participation and commitment to excellence were present.^43^

### Supporting Team Reflectivity

Innovative organisations have identified the importance of measures that facilitate reflection by team members as encouraging innovation, especially in a high-pressure environment where there is a heavy workload, tight deadlines, high expectations and a less than an optimum working environment. These innovative organisations believe that leaders/managers should be encouraged to create opportunities for team members to reflect inside and outside the traditional work environment. The experience of the 3M Company has been especially influential. Since the 1940s it has encouraged its thousands of technical employees, regardless of their roles, to use 15% of their paid weekly hours (ie, the 3M 15% Rule) to reflect/work on independent ideas. Organisations such as the 3M Company, have outperformed their competitors because the innovative ideas generated by all employees are valued, leading to better business outcomes.^44^

A study by Schippers et al^45^ found that leaders/managers who encourage team reflectivity can promote innovation in less than optimum working conditions.^45^ This cross- sectional quantitative study included 1156 members of 98 primary health teams in the United Kingdom. However, the researchers were not privy to the content of the reflective process, including: if and how often reflexive sessions took place; what was discussed; and how the discussions resulted in innovations. In addition, they could not ascertain whether prior innovation successes may have influenced the observed results.

Thus, authors have advocated reflectivity as a means of fostering improved performance, one element of which is to encourage innovation,^46^ this does not seem to have attracted much attention from empirical researchers examining leadership in the health sector.

### Employee Mindset

An Employee Mindset, with a willingness (ie, growth versus fixed orientation) to learn from errors, is promoted as supporting innovation. For example, an employee with a growth mindset generally believes it is beneficial to their career development to learn from their mistakes. In contrast, an employee with a fixed mindset may be more interested in ‘looking good’ and may avoid changing situations which may cause them to possibly make a mistake. We have included this concept because a fixed mindset has been shown to stifle innovation even where a leader exhibits a leadership style that supports innovation. However, when an employee growth mindset is combined with a leadership style where the leader/manager believes in encouraging learning and improving employees, a less risk-averse environment may be created which further supports organisational innovation. ^47,48^ There is, however, to our knowledge, little research on this specific topic in the health sector. In a quantitative study of the role mindset performed across three continents including Europe, the United States and China with 554 employees from varying economic sectors, Bligh et al, while discussing the implications of mindset and errors in the health sector, stated they “*were unable to examine the impact of industry and organizational culture”*…and “*the relationship of leadership style and error learning.”* Bligh et al, in a paper published in 2018, argued that further research is necessary.^49^

### Organisation Culture That Supports Innovation

Certain aspects of culture can facilitate transmission of messages of leaders/managers to an organisation and make them more clearly understood. Such messaging is particular effective when accompanied by rewarding individuals for risk-taking.^50^ In a quantitative study of 658 participants from Australian law firms, leaders/managers that effectively used Schein’s three cultural layers in organisational messaging (ie, setting cultural values, norms and artefacts) had a greater certainty that their communications were accepted and led to innovative behaviours.^51^ In the health sector, a study of chief information officers in German hospitals linked their ability to innovate to the presence of a “well-structured, formalised and strategy oriented environment.”^52^

Leaders that create an organisation with a culture that emphasizes success, encourages and rewards risk-taking are more likely to be successful, at all phases of the innovation process. Organisational culture in health organisations can be measured^53^ and has been linked to performance of hospitals^54^ and differences in patient outcomes.^55^ There are several studies showing how it can be changed, although these have not been linked to propensity for innovation per se.^56^

### Style of Senior Leadership

Enthusiasm, opinion leadership and vision of the Chief Executive Officer (CEO) are considered critical determinants of an organisation’s success at innovating. For example, CEO leadership drives innovation by creating an environment with high quality work relationships. An enthusiastic and visionary CEO (ie, a transformational leadership style) creates an organisational culture that encourages open knowledge integration and learning essential for innovation.^22,32,57,58^

Leadership styles have been studied extensively in many sectors, including research that has focused on innovation. For example, a quantitative study of 1340 individuals, including managing directors and CEOs from 227 professional firms and business units in six countries (including the United Kingdom and the United States), encompassed the ‘Full Range Leadership Model’ and the ‘Upper Echelons Perspective’ (ie, a focus on the influence of senior leadership in fostering innovation) and ‘Visionary Leadership’ framework (ie, the importance of senior leadership to influence innovation by articulating a credible vision-Transformational Leadership Style). The principal aim of this study was to determine whether strategic leadership influenced product or administrative innovation. The study found that strategic leadership influences innovation exclusive of the effects of organisation size and culture, among other factors.^59^

In the health sector, research on leadership styles has focused on issues such as their role as predictors of patient outcomes,^60^ avoidance of burnout,^61^ satisfaction or retention of nurses^62-64^ and other health professionals,^65^ and changes in the focus of organisations.^66^

There is rather less on leadership for innovation. A study of nurses in Iran found that entrepreneurial leadership was associated with innovative behaviour.^67^ A study undertaken among medical professors at Imperial College, London, found that leadership styles did not correlate with conventional measures of academic performance, such as publications and citations, but a transformational leadership style, characterised by idealised attributes, idealised behaviours, inspirational motivation, intellectual stimulation, and individual consideration were highly significant predictors of leadership outcomes, including effort, effectiveness, and satisfaction.^68^

## Discussion

### Key Findings

We have identified eight theories and concepts that may be useful to those charged with fostering innovation in the health sector, reviewing the experience of applying them in this particular context. Although the evidence is limited, we hope that this brief review will help those in these roles as they develop their own styles of working.

A caveat is necessary. As Creswell^69^ has noted, management theories should be predictive of phenomena (ie, health innovation in this instance). However, there is no guarantee that they will lead to the predicted results under all circumstances. Therefore, health sector leaders/managers need to exercise due diligence in the application of these leadership theories and concepts in practice. They should especially consider the organisational ‘Context’ in which these leadership theories and concepts are applied. In certain instances, leadership is the “*emergent property of the interaction between the leader/manager, the organization member and the situation”* (V. Iles, Personal Communication, May 13, 2018).

Leadership has always been important at all levels of the health sector. Similarly, health organisations have always been expected to innovate, either in terms of adopting new treatments and models of care. However, in several countries there is a growing expectation that many health organisations, such as Academic Health Science Centres do more, explicitly working to develop, evaluate, and implement innovations. Those who lead these organisations face many challenges.^70^ They must balance many competing responsibilities, combining high quality patient care with state of the art research. They must manage highly skilled, often individualistic, professionals who themselves have different agendas, not always aligned with those of the organisation. They must engage with a wide range of stakeholders, including those who pay for patient care and those who pay for research. And they must manage complex partnerships, typically spanning the public and private sectors, with the complexities that this can create.^71^ Yet while leadership in some sectors, most notably manufacturing and technology, has been the subject of an enormous volume of research, this group of leaders has, so far, received much less attention from researchers. Obviously, much research on leadership cannot simply be applied to this sector, not least because of the sheer complexity of the organisations involved. There are publications setting out proposals for how such leaders should be trained^72^ but there is much less on what works, or does not, as they seek to refine their leadership styles, their relationships with their subordinates, the culture of their organisations and much else. In the next section, we set out three areas of future research that may help to understand those factors that can support improved leadership for innovation in healthcare.

### Future Research

Our approach has allowed us to take a broad perspective of theories and concepts that may be relevant to leadership for innovation in healthcare. This seeks to overcome the challenge, for those engaged in this area, of searching through a large, but fragmented literature. However, it has also allowed us to identify some areas where future research could be useful:

1. There is a need for more research on ‘Alignment of Leadership Styles’ in the health sector, looking at how it plays out across the four innovation process phases. Concurrently, this research should include examining the leadership styles that lie on a continuum from positive to negative leadership styles including Authentic, Transformational, Transactional, Aversive or Laissez-faire organisational leadership styles.^44,47,48^ For example, creating a Psychological Climate for Innovation might be expected to encourage leaders/managers feel safe to introduce new opportunities in the ‘*Search’* phase for new ideas/opportunities as innovation is expected and rewarded. Leaders/managers therefore may have less cause to fear from internal jealousies because they are introducing an idea that others in the organisation may have overlooked in the past.
2. We believe that ‘Group Cohesiveness’ deserves further study as to its role in innovation in the health sector. Cohen and Bailey,^73^ drawing on evidence from other sectors, have noted how *“‘Group Cohesiveness’ [Emphasis added] is positively related to performance. Three meta analyses and several empirical studies found a slight to moderate positive relationship between ‘Group Cohesiveness’ and performance. This is a robust finding in an area that has long been studied.”*
3. As previously discussed, future research needs to study the role of the ‘Context’ of an organization in which the leadership theories and concepts favourable to health innovation discussed in this perspective are applied.

## Conclusion

Using a narrative approach to synthesise the literature, we have identified eight theories and concepts that may be applied by health leaders/managers to drive innovation through the four phases of the innovation process. However, as leadership of health innovation is complex and situational, we recommended threes areas for future research including examining the ‘Alignment of Leadership Styles’ operationalised with the four innovation process phases; the organisational ‘Context’; and ‘Group Cohesiveness.’

As noted previously, this review is exploratory and cannot hope to be comprehensive. For example, it is likely that there are valuable insights to be gained from, for example, biographies of those who have occupied these leadership roles but which may be difficult to find. However, while noting the limitations of this review, one thing can be said with confidence. The importance of leadership for innovation in the health sector will increase but, so far, there is only limited research to guide this process. Our hope that this review will encourage future research including the areas proposed in this perspective.

## Ethical issues

Not applicable.

## Competing interests

Authors declare that they have no competing interests.

## Authors’ contributions

PW drafted the paper and MM revised it.

## Supplementary files

Supplementary file 1 contains literature review methods.
