# Supplementary material for: Leadership for Innovation in Healthcare: An Exploration
Source: Int J Health Policy Manag. 2018 Dec 17;8(3):138–44. doi: 10.15171/ijhpm.2018.122 (PMC6462199; doi:10.15171/ijhpm.2018.122)
Supplement: Supplementary file 1 — contains literature review methods. [file ijhpm-8-138-s001.pdf]

# Supplementary File 1

## Literature Review Methods

A literature review was performed to identify studies that had examined leadership and innovation in the health and other sectors and to identify empirically supported concepts and theories that should promote health innovation. The results of the literature review were used to gain a better understanding of the 'Context' with respect to leadership of health-related research and development activities within the UK and US. In addition, the literature review performed through January 2018 was instrumental in structuring a survey questionnaire and initial interview questions used for a more comprehensive research study, *Weintraub P. Leadership of Health Innovation: Building an Innovative health Organisation (A Mixed-Methods Study).*[74] As depicted in Figure 1, the final sample framework for this research study included 155 participants from 8 health organisations in the UK and the US.

**Figure 1.** Research Study Sampling Framework.

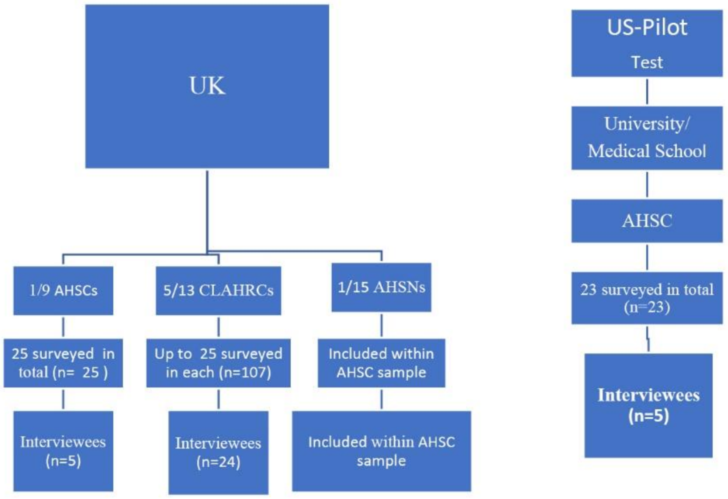


Information was extracted from the papers reviewed by applying the PEO/PIO (Population, Exposure [Issue], Outcome) or PICO (Population, Patient, Problem, Intervention, Comparison, Outcome) structure as appropriate for the type of study to identify studies of the leadership styles of health leaders/managers that are most likely to drive much needed health innovation. A keyword search string was prepared using a selection of electronic databases, including MEDLINE, PsycEXTRA, Econlit, and Embase. In addition, an Internet search using the Google search engine was performed to identify any documents published online by a government agency or other organisations providing relevant information and to update the results of earlier searches. Also, extensive hand searching of references was performed in papers identified in the searches described above (articles/studies) even if those source

papers were subsequently excluded from this review. The specific inclusion and exclusion criteria used in the review are summarized in Table 1.

**Table 1.** Specific Inclusion and Exclusion Criteria

| **Inclusion** | **Exclusion** |
|---|---|
| 1. Published articles/books/studies providing information and evidence relevant to leadership styles and characteristics of leaders/managers most likely to drive health innovation. | 3. Published articles/books/studies or government/other organisation documents that are not specifically addressing the leadership/management of innovation |
| 2. Articles/studies/documents published solely online by a government agency or other organisation providing information and relevant evidence. | 4. Published articles/books/studies or government/other organisation documents that are not specifically addressing a subject relevant to the leadership of innovation or excluding mention of innovation altogether |

No published or other articles/studies were excluded from the three searches based upon the methodology, published date, geographical location or language of journal publication. Quality assessment of the papers included was performed using the Critical Appraisal Questionnaire developed by the Centre for Evidence-Based Management.[75] This is similar to other widely recognized tools for the assessment of qualitative and other studies.

**Synthesis of Extracted Evidence**

To perform the literature review, the Matrix Method adapted from Garrard[76] was applied. As used, this had four components, including a:

1.) Paper Trail Folder-to document the process of undertaking the literature review;

2.) Documents Folder-for retaining documents for review;

3.) Review Matrix Folder-a spreadsheet format used to abstract each document included in the thesis; and a

4.) Synthesis Folder- this folder is an expansion of the preceding spreadsheet where I commented on each reference. As described previously, the quality of references in this thesis was assessed using a standardized tool. [75] The headings of this spreadsheet are illustrated in Table 2.

**Table 2.** Illustration of Matrix Method Spreadsheet

| Author, Title, Journal | Year Published | Purpose | Type of Study | Study Themes | Study Questions-Theory | Literature Review Comments-Narrative Synthesis | Hypothesis Tested-Including sample framework |
|---|---|---|---|---|---|---|---|

Source: Adapted from Garrard[76]

A narrative synthesis approach was used to synthesize the data extracted from the selected studies. The objective of this approach was to give more weight to the collective synthesis of the selected studies than would be achieved with an analysis solely based on a singular view of each study (ie, the sum of the parts [of the individual studies selected] would contribute more, collectively, to the questions posed than the studies read and analyzed singly).[21]

**Results of the Literature Review**

As depicted in the Figure 2, a total of 3,643 results were obtained in the four phases of electronic data searches using the Ovid search engine. The first phase used the search string of innovation* and health* and networks* and United Kingdom. This yielded 840 results, net of 19 duplicates. The second search phase used the search string of innovation* and health* and networks* and leadership*. This phase yielded 2,660 results, net of 38 duplicates. The third search phase used the search string combining innovation* and health* and leadership* and the search string of health* and leadership styles* and characteristics*. This yielded 64 results. The final and fourth phase used the search string of Social Capital* and health leaders* and yielded 17 results, net of 5 duplicates. An additional 105 books/articles/publications were obtained from hand/Google Internet engine searching of references to the studies where the full text was assessed. After reviewing the abstract of the records or summary of the records, a total of 3,467 records were excluded as not relevant to this research study. A total of 219 full-text records were read and analyzed and 109 of these records were excluded based on the criteria in Table 1. This resulted in 110 records that were included in the reference list for the research study, *Weintraub P. Leadership of Health Innovation: Building an Innovative health Organisation (A Mixed-Methods Study).*[74]

**Figure 2.** PRISMA Flow Diagram.

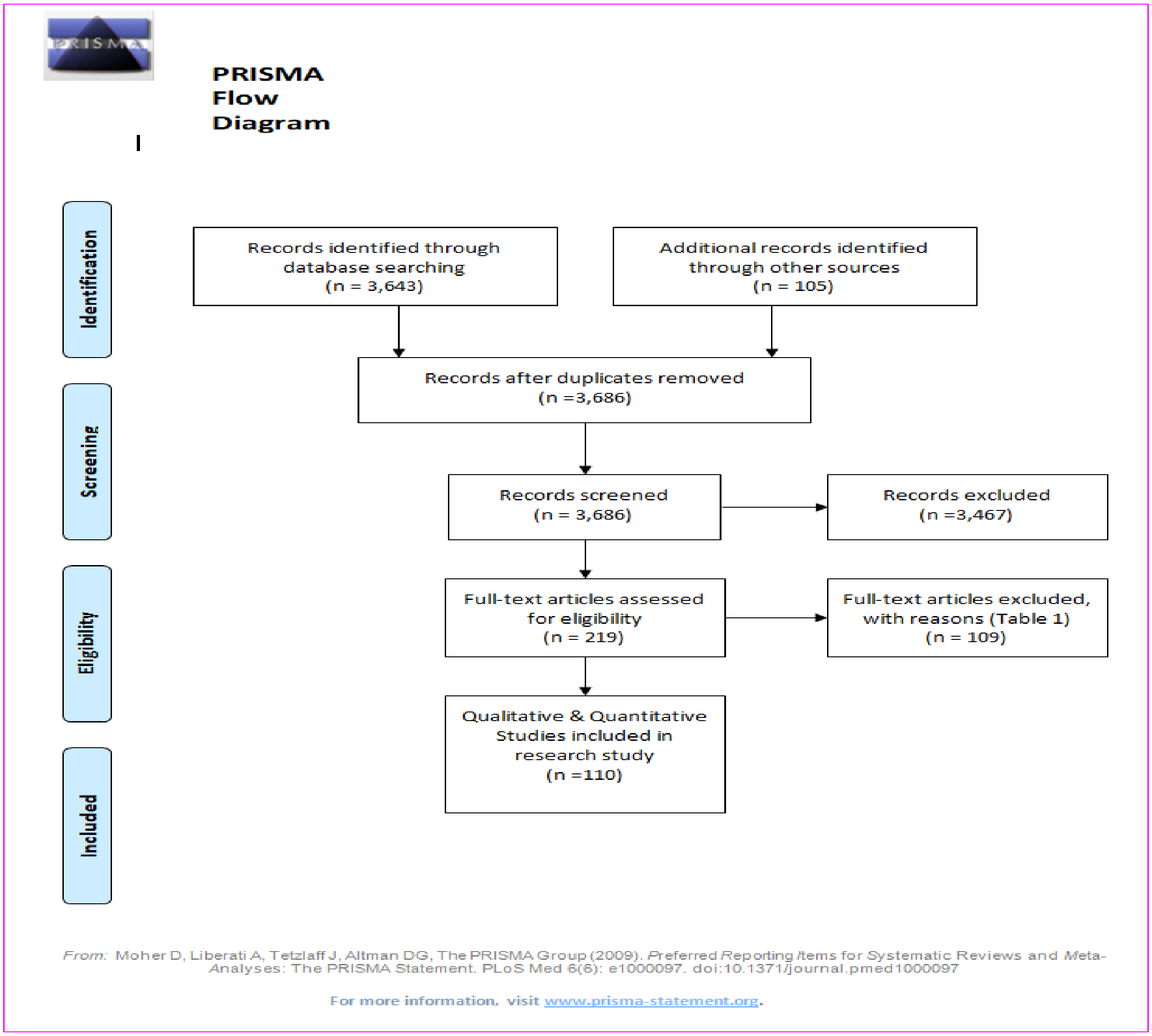


Only those leadership theories and concepts proposed as drivers of health innovation were included if they were supported by empirical research. Others, which were more speculative, proposing but not testing theories and concepts, were excluded. In addition, an Internet search using the Google search engine was performed to identify any documents published to update the results of the earlier searches depicted in Figure 2 through the date of submission of this perspective. Also, extensive hand searching of references was performed in papers identified in the searches described above (articles/studies) even if those source papers were subsequently excluded from this review for the purposes of responding to the narrower research question posed in this perspective.
